# Long-Term Follow-Up of Professional Soccer Players: The Analyses of Left and Right Heart Morphology and Function by Conventional, Three-Dimensional, and Deformation Analyses

**DOI:** 10.3390/diagnostics15141745

**Published:** 2025-07-09

**Authors:** Joscha Kandels, Michael Metze, Stephan Stöbe, Lisa Do, Maximilian Nicolas Möbius-Winkler, Marios Antoniadis, Andreas Hagendorff, Robert Percy Marshall

**Affiliations:** 1 Klinik und Poliklinik für Kardiologie, Universitätsklinikum Leipzig, 04103 Leipzig, Germany; 2 RasenBallsport Leipzig GmbH, Cottaweg 3, 04177 Leipzig, Germany; 3 Department of Orthopedic and Trauma Surgery, Martin-Luther-University Halle-Wittenberg, 06120 Halle, Germany

**Keywords:** athletes, transthoracic echocardiography, long term follow-up, soccer, athlete’s heart, strain, deformation imaging, speckle tracking, 3D echocardiography

## Abstract

**Background:** Transthoracic echocardiography (TTE) is the primary imaging modality to assess cardiac morphology and function. In athletes, distinguishing physiological adaptations from pathological changes is essential. This study aimed to evaluate long-term cardiac structural and functional changes in professional soccer players. **Methods:** This retrospective study included 20 healthy male professional soccer players (mean age 21.2 ± 3.4 years) from the German first division, examined annually from 2016 to 2024 (mean follow-up 5.6 ± 2.0 years). TTE parameters associated with the “athlete’s heart” were assessed, including left ventricular end-diastolic diameter (LVEDD), interventricular septal thickness (IVSD), relative wall thickness (RWT), indexed LV mass (LVMi), and left atrial volume index (LAVi), along with 3D-derived LV and RV volumes. Advanced deformation imaging included global longitudinal strain (GLS), right ventricular strain (RVS), and left/right atrial reservoir strain (LASr and RASr, respectively). Baseline and final follow-up values were compared. **Results:** No significant changes were observed over time in conventional or advanced echocardiographic parameters (e.g., LVEDD: 54.5 ± 3.1 mm vs. 54.6 ± 3.9 mm; *p* = 0.868; GLS: −18.7% ± 2.2% vs. −18.4% ± 1.9%; *p* = 0.670). Ventricular volumes and strain values also remained stable throughout follow-up. **Conclusions:** Over a mean follow-up of more than five years, professional soccer players showed stable cardiac morphology and function without evidence of pathological remodeling. These findings support the concept that long-term high-level training in mixed-discipline sports leads to balanced, physiological cardiac adaptation.

## 1. Introduction

Among the various imaging modalities available, transthoracic echocardiography (TTE) plays a central role in the assessment of cardiac morphology and function as a cost-effective and widely available imaging modality that provides non-invasive insights into structural and functional adaptations, such as wall thickness and cardiac mass and volumes. Three-dimensional (3D) echocardiographic measurement of cardiac volume and function has proven to be an excellent tool for accurately detecting changes over time in healthy subjects [1]. Deformation imaging based on speckle-tracking echocardiography (STE) is a relatively new imaging modality which can provide further insights in the athlete’s heart [2]. Previous studies have shown that STE allows for early detection of myocardial changes, e.g., due to myocardial fibrosis [3,4,5].

The echocardiographic changes most frequently described in athletes include left ventricular hypertrophy (LVH), increased LV end-diastolic diameter (LVEDD), and augmented left atrial (LA) size. These changes are typically linked to an increased stroke volume (SV) and cardiac output, which, in turn, enhance cardiovascular performance. The definition of an athlete’s heart in echocardiographic terms is centered on these structural and functional adaptations, which vary depending on the type and intensity of training. Endurance athletes, such as long-distance runners and cyclists, tend to exhibit eccentric LV remodeling, characterized by augmented chamber size and preserved systolic function. Recognizing these patterns is essential to ensure that athletes receive appropriate medical care and that a balance between performance optimization and cardiovascular safety is maintained [6].

The cardiovascular assessment of professional athletes constitutes an important component of pre-participation screening and routine medical evaluations. Differentiating benign adaptations from pathological conditions, such as hypertrophic cardiomyopathy or arrhythmogenic right ventricular cardiomyopathy, is a major challenge in sports medicine [7]. Due to the intense physical demands that professional athletes are exposed to, comprehensive diagnostic approaches to the cardiovascular system are necessary to differentiate physiological adaptations from pathological conditions that may predispose athletes to sudden cardiac events [8].

There is a lack of data on professional soccer players who have been followed up over a long period of time by basic and modern echocardiographic parameters. The objective of the present study was to analyze basic and advanced echocardiographic parameters over several years in professional athletes to elucidate the spectrum of cardiac adaptations and to contribute to the refinement of criteria for distinguishing physiological adaptation from pathological abnormalities.

## 2. Materials and Methods

### 2.1. Study Population and Study Design

The study included 20 soccer players from the first soccer division in Germany. All athletes provided informed consent after a full explanation of the purpose and order of all procedures. The study was conducted in accordance with the Declaration of Helsinki and was approved by the ethical committee of the University of Leipzig (073/18-ek).

All athletes were enrolled in the outpatient clinic of cardiology from July 2016 until July 2024. All athletes were asymptomatic and completely free of cardiovascular diseases or risk factors. A physical examination including vital parameters was performed in all athletes. Not all of the soccer players underwent an echocardiographic examination every year because some joined or left the team during the follow-up period, resulting in an inconsistent number of measurements each year.

### 2.2. Tracking the Seasonal Load Data

The collection of seasonal external load data was conducted using the Apex Pro System (sampling rates: GPS—18 Hz, GNSS—10 Hz, triaxial accelerometer—952 Hz, gyroscope—952 Hz, magnetometer—10 Hz; STATSports Group Limited (Newry, North Ireland)). The team’s home match data were subjected to optical analysis using the Chiron Hego system (Chiron Hego, Cologne, Germany). The respective national teams provided data on additional national-team camps and matches. Data were collected on the total number of loading days and the total loading time as a combination of all trainings and matches (in minutes and days). Regarding physical metrics, we collected data on total running distance (in km) and training intensity as measures of high-speed running. The latter was calculated as high metabolic load distance (HMLD), which was previously defined as the distance covered with a metabolic power of at least 25.5 watt/kg, i.e., with speed > 5.5 m/s and acceleration/deceleration > 2 m/s [9,10]. Goalkeepers were excluded from the load analysis because there was inadequate documentation of their training loads.

### 2.3. Transthoracic Echocardiography

TTE was performed in all athletes using a Vivid E9 or E95 ultrasound system with a 4Vc phased-array probe (GE Healthcare Vingmed Ultrasound AS, Horten, Norway). EchoPac software (Version 206, GE Healthcare Vingmed Ultrasound AS, Horten, Norway) was used for post-processing analyses. All deformation measurements were conducted using the same automated software (EchoPAC software version 206, GE Healthcare).

### 2.4. Left Heart Morphology and Function

**Figure 1:**
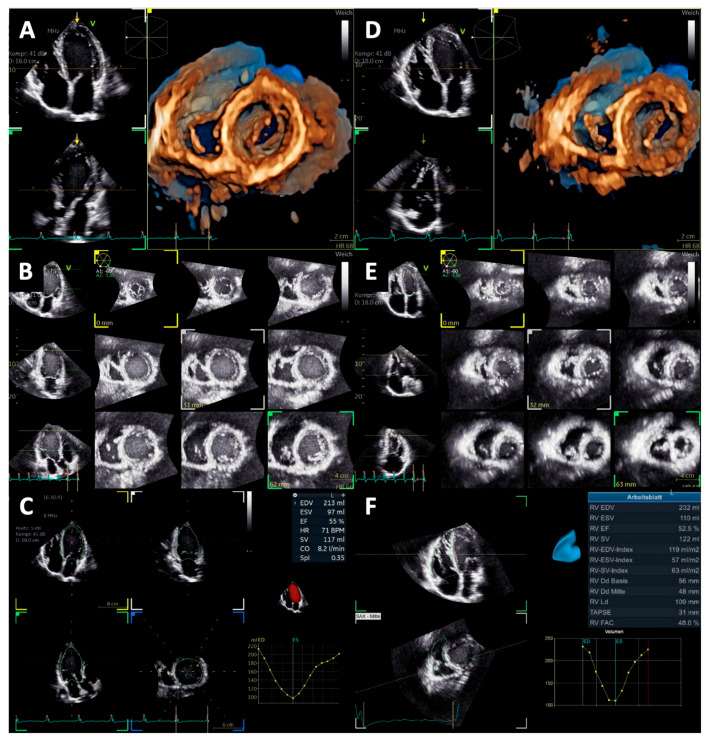
Documentation of the quality standard for volume measurements of the left and right ventricle (LV and RV, respectively). (**A**) 3D LV short-axis view, (**B**) LV multi-slice view, (**C**) LV “beutel” volume measurement, (**D**) 3D RV short-axis view, (**E**) RV multi-slice view, and (**F**) RV “beutel” volume measurement.

The diameter of the LV outflow tract (D_LVOT_) was measured in the left parasternal long-axis view. Relative wall thickness (RWT) was calculated as twice that of the LV posterior wall thickness at the end-diastole (LVPWD) divided by the LVEDD. Measurements of cardiac dimensions were performed using the anatomical M-mode in the parasternal long-axis view, while standardization was verified by biplane imaging. LV mass (LVM) and LV mass index (LVMi) were calculated using the Devereux formula according to current guidelines. Normal RWT was defined as ≤0.42 and normal LVMi was defined as ≤115 g/m^2^. Using RWT and LVMi, LV geometry was classified into four groups, nMWLT normal LV geometry (RWT ≤ 0.42 and normal LVMi), eccentric LVH (RWT ≤ 0.42 and increased LVMi), concentric LV remodeling (RWT > 0.42 and normal LVMi), and concentric LVH (RWT > 0.42 and increased LVMi) [11]. LV systolic function was assessed by LV ejection fraction (LVEF), determined from the end-diastolic (LVEDV) and end-systolic volume (LVESV) using LV biplane planimetry based on the modified Simpson’s rule in the apical two- and four-chamber view. The LV stroke volume (LVSV Doppler) was also determined using the Doppler method, and the cardiac index was calculated by multiplying the indexed stroke volume by the heart rate [11]. In addition, 3D LV volumetry was performed according to current recommendations to assess LVEDV and LVESV [11], as shown in Figure 1. Automated detection of endocardial contours was verified and, if necessary, was manually adjusted by the user.

**Figure 2:**
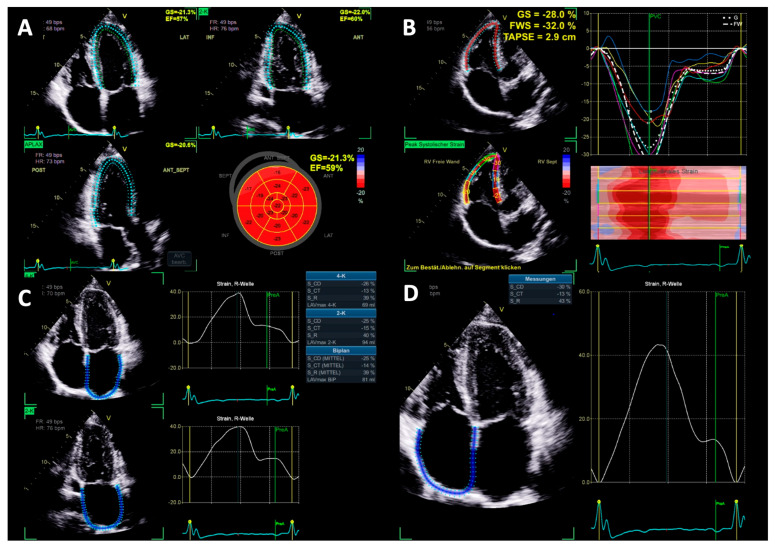
Documentation of the quality standard for cardiac chamber deformation measurements (LV = left ventricle, RV = right ventricle, LA = left atrium, and RA = right atrium). (**A**) Triplane LV global longitudinal strain measurement, (**B**) RV free wall strain, (**C**) LA biplane strain measurement, and (**D**) RA monoplane strain measurement.

LV deformation was assessed by global longitudinal strain (GLS) based on two-dimensional (2D) STE in the apical long-axis, two- and four-chamber view according to current guidelines (Figure 2) [12]. As with 3D volumetry, endocardial contours and regions of interest were verified and manually adjusted by the user if necessary. Only segments with reliable tracking were considered [12]. In addition, left atrial strain (LAS) was measured in the apical two- and four-chamber view following the recommendations of the European Association of Cardiovascular Imaging (EACVI) [12] and adjusted, as with the previous measurements (Figure 2). The reference point was set at the end of LV diastole, identified by transmitral inflow. The three phases of LAS were classified as follows: LA reservoir strain (LASr)—peak strain before mitral valve opening during LV systole; LA contractile strain (LASct)—strain component at the end of LV diastole minus the strain at the onset of atrial contraction; and LA conduit strain (LAScd)—difference between LASct and LASr. Further, maximum left atrial volume (LA Vol_max_) was determined automatically based on the delineation of the atrial border and was indexed (LAVi) to body surface area (BSA). For each parameter, mean values based on apical two- and four-chamber view measurements were considered. [13].

Diastolic function was assessed by measuring the maximum blood flow velocities (V_max_) of the E- and A-wave, the E/A ratio, myocardial V_max_ of e’ and a’ at the basal septal and lateral mitral annulus, the septal and lateral E/e’ ratio, and the systolic pulmonary artery pressure (sPAP) according to current guidelines [14].

### 2.5. Right Heart Morphology and Function

Right ventricular (RV) EDV, ESV, and SV were measured by 3D volumetry in the apical four-chamber view, as displayed in Figure 1. In addition, RVSV was assessed by Doppler echocardiography. RV free wall strain was assessed in the focused apical four-chamber view according to current guidelines [13]. The region of interest was set at the endocardial border and was manually adjusted if necessary. Furthermore, right atrial (RA) strain was measured in the apical four-chamber view, like the LA strain, by automated contour detection by the software and was adjusted manually if necessary (Figure 2). The measurement was repeated three times, and an average value was calculated as recommended [13]. Also, the maximum right atrial volume (RA Vol_max_) was assessed automatically by the software based on the delineation of the atrial wall and was indexed to BSA (RAVi).

### 2.6. Statistical Analysis

All statistical analyses were performed using SPSS Statistics (version 24.0, IBM, Armonk, NY, USA) and Microsoft Office Excel (version 16.94, Microsoft, Redmond, WA, USA). Continuous variables were expressed as mean value ± standard deviation (SD). In consideration of the small sample size, we decided to forgo distribution analyses. Statistical significance was accepted for a *p* value < 0.05. The Student’s *t*-test was used to compare the echocardiographic results at baseline and end of follow-up. The Kappa coefficient (κ) was used for the assessment of intra- and interobserver variability in 20 athletes under identical conditions. An independent examiner, blinded to the initial results, performed the second evaluation.

## 3. Results

The baseline characteristics are shown in Table 1. None of the players had a positive family history of sudden cardiac death. A median of six echocardiographic examinations was performed on each player during the follow-up period.

Total training days and time ranged from 126 to 256 days (mean 201 ± 17 days) and 10,597 to 20,400 min (mean 12,283 ± 3074 min) per year. Total distance and HMLD covered ranged from 668.541 to 1,146,378 m (mean 703,704 ± 345,343 m) and 81,462 to 194,464 m (mean 128,930 m ± 33,703 m) per year.

Conventional echocardiographic parameters, such as LVEDD (54.5 mm ± 3.1 vs. 54.6 mm ± 3.9; *p* = 0.868), diastolic interventricular septal thickness (IVSD) (9.9 mm ± 1.0 vs. 9.9 mm ± 1.2; *p* = 0.878), RWT (0.35 ± 0.05 vs. 0.36 ± 0.09; *p* = 0.539), LVMi (104.8 mg/m^2^ ± 16.6 vs. 101.5 mg/m^2^ ± 17.5), and LAVi (29.8 mL/m^2^ ± 5.2 vs. 31.1 mL/m^2^ ± 7.4; *p* = 0.401) did not differ between baseline and final follow-up. Significant changes could be observed in LVEF_biplane_ (Table 2), E/A-ratio (Table 3), and LV- (Table 2) and RVSV_Doppler_ (Table 4). At baseline. two athletes presented a mildly reduced LVEF, which is displayed in Figure 2.

Initially, 15 out of 20 athletes had normal LV morphology, 3 demonstrated eccentric LVH, and 2 demonstrated concentric LVH according to current guidelines [11]. At follow-up, 14 athletes demonstrated normal LV morphology, 2 athletes demonstrated eccentric LVH, 2 athletes demonstrated concentric LVH, and 2 athletes demonstrated concentric LV remodeling. All athletes exhibiting concentric LVH were found to have African roots.

LV- and RVSV assessed by Doppler echocardiography were significantly higher at final follow-up compared to baseline. Nevertheless, there were no significant differences between LV- and RVSV at baseline compared to final follow-up when assessed by 3D volumetry (Table 4). Despite the absence of statistical significance, LV- and RVEDV assessed by 3D volumetry increased over time, resulting in increased LV and RV stroke volumes. LVEF by 2D biplane volumetry was significantly higher at final follow-up compared to baseline, whereas LVEF assessed by 3D volumetry did not differ significantly (Table 2 and Table 4). RVEF was in normal ranges at baseline and at final follow-up (Table 4).

**Figure 3:**
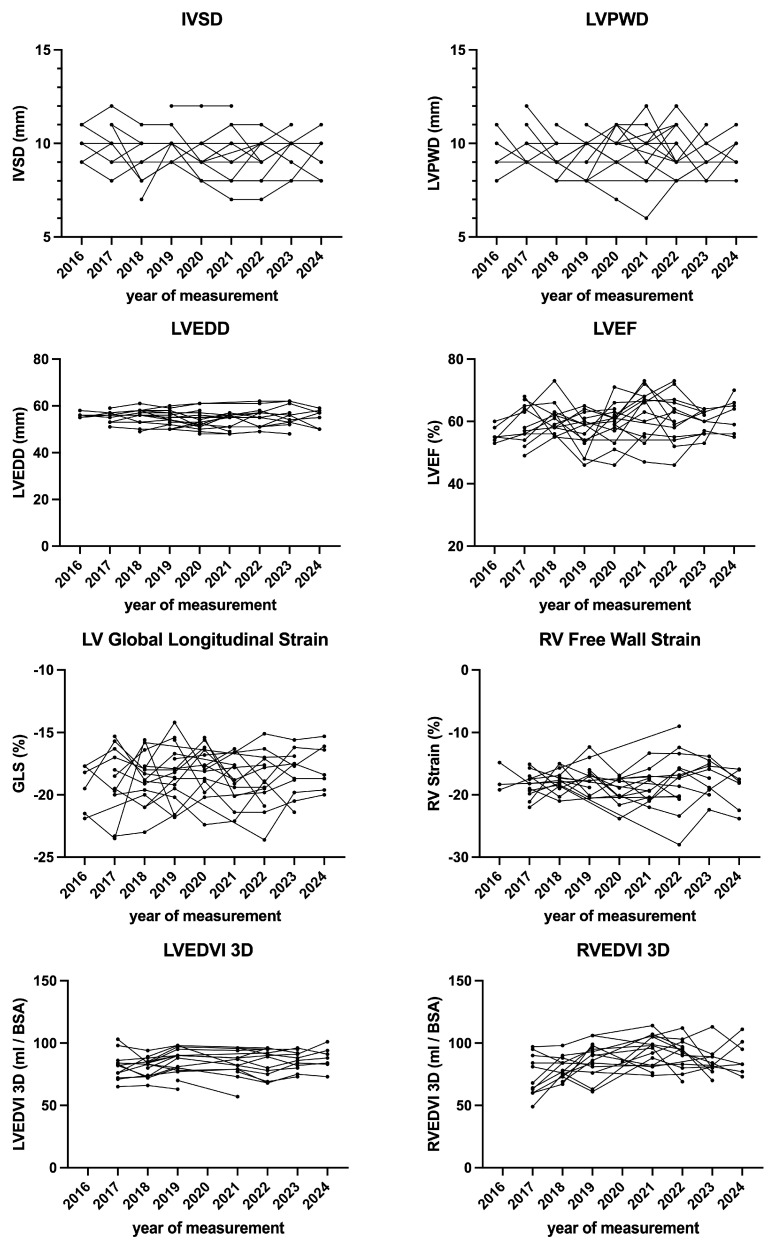
Echocardiographic parameters of ventricular morphology and function over time.

**Figure 4:**
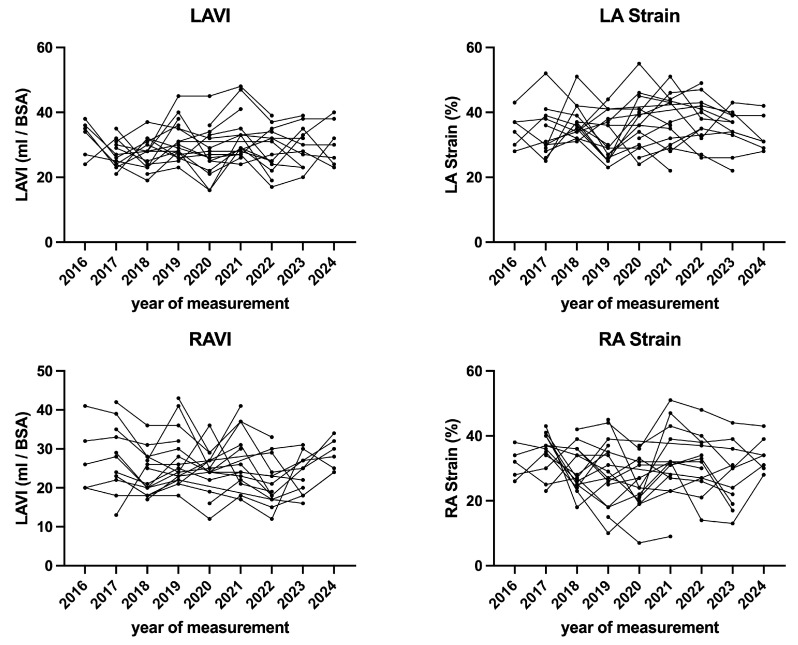
Echocardiographic parameters of atrial morphology and function over time.

GLS (Table 2), LA strain (Table 3), RV free wall strain (Table 4), and RA Strain (Table 4) did not differ significantly between baseline and final follow-up. Strain values of left and right ventricle over the time are displayed in Figure 3, whereas left and right atrial strain parameters are displayed in Figure 4.

The intra-observer variability showed high agreement for LVEF biplane (κ = 0.86; z = 4.37, *p* < 0.001), GLS (κ = 0.89; z = 4.45, *p* < 0.001), RV Strain (κ = 0.90; z = 4.61, *p* < 0.001), LA Strain (κ = 0.89; z = 4.44, *p* < 0.001) and RA Strain (κ = 0.87; z = 4.39, *p* < 0.001). In addition, interobserver variability between two investigators also showed good agreement for LVEF biplane (κ = 0.72; z = 4.28, *p* < 0.001), GLS (κ = 0.80; z = 4.44, *p* < 0.001), RV Strain (κ = 0.87; z = 4.58, *p* < 0.001), LA Strain (κ = 0.76; z = 4.33, *p* < 0.001), and RA Strain (κ = 0.75; z = 4.44, *p* < 0.001). Intra- and interobserver variability for the remaining echocardiographic measurements also consistently demonstrated good agreement.

## 4. Discussion

In the present study, conventional and advanced echocardiographic parameters of left and right heart morphology and function were assessed over a period of up to nine years in 20 professional soccer players.

The main findings of the present study are as follows:

(1) Most of the athletes showed normal LV and RV morphology and function at baseline, despite all athletes playing professional soccer for many years.
(2) Neither conventional nor advanced echocardiographic parameters indicated the development of an athlete’s heart in this cohort.

### 4.1. Introduction to Cardiovascular Adaptations

Cardiac adaptations in professional athletes are influenced by several factors, such as sporting discipline, ethnicity, age, body size, and gender, as well as drugs and cardiomyopathies. Pelliccia et al. examined 1309 elite athletes aged 13–59 years and found that LV cavity dimensions varied widely. Notably, approximately 15% of participants exhibited LV enlargement to a degree compatible with primary dilated cardiomyopathy. However, systolic function remained normal, suggesting that this may represent a physiological adaptation to intensive training rather than a pathological condition [15]. In contrast, 55% of athletes demonstrated LV cavity dimensions within normal limits. Subsequent work by Caselli et al., which used 3D echocardiography in 511 Olympic athletes, confirmed a balanced adaptation of LV volume and mass, with preserved systolic function, regardless of the athletes’ sport discipline [16]. RV remodeling has also been studied in athletes. Zaidi et al. evaluated RV characteristics in 300 Black athletes and found that both Black and white athletes had larger RV dimensions than sedentary controls. In addition, body size is another major determinant of cardiac morphology, explaining nearly 50% of the variability in LV cavity size and mass among athletes [17].

LA enlargement has been observed as part of the athlete’s heart. Pelliccia et al. reported LA enlargement (anteroposterior diameter ≥40 mm) in 20% of 1777 competitive athletes, but found that supraventricular tachyarrhythmias—including atrial fibrillation—were rare (<1%) and unrelated to LA size [18]. This supports the concept of benign LA remodeling in athletes [18]. Ethnicity is another important factor. Rawlins et al. compared 240 Black and 200 white female athletes, finding that Black athletes had more pronounced LV hypertrophy and more frequent repolarization changes [19]. Di Paolo et al. confirmed similar ethnic differences in a male soccer player cohort [20].

In addition to the previously mentioned intrinsic factors, sporting discipline plays a critical role in determining the pattern and magnitude of cardiac adaptation. This relationship is driven by a dose–response effect between the intensity, duration, and nature of training and the degree of cardiac remodeling [21]. Professional athletes typically engage in structured training programs of 10–15 h per week depending on their discipline [8]. Traditionally, sports were classified by their predominant hemodynamic load as either isotonic (dynamic) or isometric (static). Isotonic training, typical of endurance disciplines, such as rowing or cycling, leads to volume overload and results in eccentric LVH. Conversely, isometric training, as seen in strength sports, like weightlifting, induces pressure overload with relatively mild wall thickening and only slight chamber dilation. However, because most sports involve varying proportions of dynamic and static components, this binary classification has proven insufficient. A more nuanced approach distinguishes between endurance, power, skill, and mixed disciplines, with mixed sports, such as soccer producing moderate increases in LV dimensions and mass. Importantly, physiological remodeling is usually harmonic, involving proportional changes across all cardiac chambers. Disproportionate or asymmetric remodeling, however, may indicate pathology [22].

### 4.2. Expected Adaptations in Soccer Athletes

Soccer is a quintessential “mixed discipline” sport, combining substantial dynamic (isotonic) and static (isometric) components. According to the classification by Mitchell et al., soccer imposes moderate-to-high levels of both isotonic and isometric loads, resulting in an intermediate degree of cardiac remodeling [23]. Mixed sports, like soccer, typically cause balanced cardiac remodeling. This involves moderate enlargement of the LV cavity and mild LV wall thickening due to combined volume and pressure loads [8,24]. Soccer players show adaptations between those of endurance athletes—who often develop eccentric LVH with large chamber dilation—and strength athletes, who tend to have concentric remodeling with thicker walls but small cavities [25,26].

In our cohort of professional male soccer players, LV morphology and function remained stable, with no significant changes in 3D LV or RV volumes, LVMi, RWT, or GLS. However, we observed a small but significant increase in Doppler-derived LVSV, likely reflecting a physiological enhancement in cardiac output. All deformation parameters remained within normal ranges, supporting preserved myocardial function. Importantly, the right ventricle followed a similarly stable pattern: 3D volumetry revealed no significant increase in RVEDV or RVESV, and RV free wall strain remained unchanged over time. Although Doppler-derived RVSV increased modestly, this was not paralleled by volumetric changes, suggesting improved dynamic output rather than structural remodeling. These findings are consistent with the notion that in mixed sports, RV adaptation is proportional and harmonic, differing from endurance disciplines in which RV cavity enlargement can be more pronounced [27].

Notably, concentric LVH was observed exclusively in players of African descent, consistent with known ethnic differences in remodeling. Previous studies have demonstrated that Black athletes tend to develop greater LV wall thickness for a given cavity size and show a higher prevalence of repolarization changes—considered a physiologic variant of the athlete’s heart [19]. The term “long-term” in the context of professional sports remains ambiguous and lacks a universally accepted definition. However, a recent study found that the average career length of professional outfield soccer players is approximately 8.6 years, with a standard deviation of 6.2 years [28]. When considered collectively, the findings from our study demonstrate that prolonged exposure to varied training stimuli in soccer results in physiological, harmonic cardiac remodeling that does not progress to a pathological phenotype.

### 4.3. Changes in Myocardial Deformation

In the present cohort, GLS, LAS, and RV free wall strain showed no alterations. These stable strain indices in high-performance athletes are consistent with published reference ranges for physiological adaptation. Caselli et al. found a mean LV GLS of 18.1 ± 2.2% in Olympic athletes compared to 19.4 ± 2.3% in sedentary controls [29]. The distribution of these values was within the normal population range [11]. The recent EAPC/EACVI guidelines similarly emphasize that athletes generally demonstrate normal GLS (usually more negative than −15%), and recommended further cardiac evaluation if the GLS is below this threshold [8]. In a similar vein, reported RV free-wall strain measurements in healthy individuals have been documented to range from −25% to −30% [30]. Furthermore, speckle-tracking studies conducted on athletes have revealed RV-GLS measurements within this normal range (e.g., ~−23% in endurance-trained soccer players and controls [31]). This phenomenon could be substantiated by an analysis of our cohort of soccer players.

Conversely, pathological states, such as hypertrophic cardiomyopathy (HCM), show a significant reduction in myocardial strain. Rich et al. found that professional soccer players had only mildly attenuated LV longitudinal strain compared to controls, with higher LV radial and circumferential strain. LV GLS in these athletes was significantly higher (more negative) than in HCM patients [32]. Persistently normal LV and RV strain, unchanged atrial strain, and preserved ejection fraction over time strongly support benign, “harmonic” remodeling consistent with classic athlete’s heart, effectively ruling out subclinical cardiomyopathy. Pelliccia et al. confirmed that preserved deformation parameters indicate physiological adaptation, whereas true cardiomyopathy shows reduced strain [8].

### 4.4. Atrial Function

In our cohort of professional soccer players, atrial size and function remained remarkably stable over a 5.6-year follow-up period. LAVi increased only marginally but non-significantly (29.8 ± 5.2 vs. 31.1 ± 7.4mL/m^2^) and LASr remained essentially unchanged. The mean LAVi (~3 mL/m^2^) observed in our athletes is well within the reference ranges for trained individuals and remains well below pathologic thresholds. D’Andrea et al. reported that highly trained athletes usually exhibit LAVi values between 26 and 36mL/m^2^, reflecting physiologic remodeling without pathological dilation [22]. In contrast, Park et al. identified LA enlargement (LAVi > 42mL/m^2^) in 19.1% of university athletes, with strong associations to training volume, cardiovascular demand of the sport, and reduced heart rate [33].

Similarly, our LASr values (mean 32–34%) remained stable and clearly above the abnormal threshold of 27.6% proposed by Park et al. in a cohort of over 1000 competitive athletes [33]. Donal et al. have emphasized that LASr is a sensitive and early marker of atrial dysfunction and may precede detectable enlargement or symptom onset [34]. Our findings of preserved strain values argue, therefore, against subclinical atrial pathology. Supporting this, Trivedi et al. demonstrated that endurance athletes with AF exhibit reduced LA strain and impaired emptying, even in the presence of normal diastolic function, suggesting early atrial myopathy [35]. In contrast, our players—young, elite, and with moderate cumulative training exposure—showed no signs of such maladaptive remodeling.

Until now, there has been a lack of evidence regarding RA in professional footballers. Nevertheless, Krittanawong sought to establish reference values for RA deformation parameters. They compared 21 studies involving 4111 healthy subjects, finding an average RA reservoir strain of 44% (95% CI: 25–63%), a contractile strain of 17% (95% CI: 2–32%), and a conduit strain of 18% (95% CI: 7–28%). It should be noted that there was significant between-study heterogeneity and inconsistency [36]. In summary, the reported values were consistent with the results of our study.

### 4.5. Differentiating Physiological Remodeling from Pathological Adaptation

Distinguishing physiological remodeling from the early stages of cardiomyopathies is a central and nuanced challenge in sports cardiology. LV geometry is usually normal or eccentrically remodeled, with RWT remaining < 0.42 in most athletes. Conversely, pathological adaptation often shows disproportionate changes, such as concentric LVH, asymmetric septal thickening, or disproportionate atrial enlargement, with impaired myocardial mechanics. This includes a reduction in GLS or LASr. A current consensus statement identifies several “red flags” for pathology, including maximal LV wall thickness > 16 mm in white athletes (or >13 mm in females), LV cavity diameters < 54 mm in the presence of marked LVH, abnormal electrocardiogram (ECG) patterns, such as lateral T-wave inversions, or a family history of sudden cardiac death [8]. The British Society of Echocardiography guideline provides more detail, recommending careful evaluation of RV/LV ratios, atrial size in relation to ventricular dimensions, and the use of 3D echocardiography and deformation imaging to identify functional abnormalities. For instance, an RVOT/RV diameter ratio > 1.0 or a GLS < −16% in a structurally normal heart may require further evaluation [37]. In uncertain cases, advanced imaging techniques, such as cardiac magnetic resonance imaging, can clarify borderline findings and improve tissue characterization. Additionally, longitudinal follow-up with serial imaging can help to distinguish between stable physiological remodeling and progressive pathology. Functional tests, such as exercise stress echocardiography, spiroergometry, or monitoring of deconditioning responses (e.g., regression of chamber size during training pauses or seasonal pauses), may provide further diagnostic information.

One challenge is distinguishing physiological cardiac adaptation in athletes from pathological conditions, such as hypertrophic cardiomyopathy (HCM) and arrhythmogenic right ventricular cardiomyopathy (ARVC). The 12-lead ECG is central to this differentiation. In HCM, findings, like deep Q-waves, diffuse T-wave inversions beyond lead V2, or significant ST-segment depression, raise suspicions of pathology. The implementation of revised criteria has enhanced the precision of differentiating between HCM and physiological hypertrophy without compromising sensitivity [38]. In the context of ARVC, the presence of precordial T-wave inversions beyond V3, low QRS voltages, and the occurrence of frequent or atypical premature ventricular contractions (PVCs) serve as significant markers [39,40]. Furthermore, the incorporation of novel ECG indices, such as the RV1-V3 transition ratio, has been demonstrated to enhance the diagnostic accuracy of differentiating the origin of PVCs [41].

### 4.6. Limitations

This study has several limitations. First, the sample size was relatively small and limited to a homogenous cohort of young, male, professional soccer players, which restricts the generalizability of our findings to other athletic populations. Second, although deformation imaging provides sensitive insights into myocardial mechanics, we did not incorporate complementary modalities, such as CMR, which remains the gold standard for tissue characterization and could have validated or enhanced our echocardiographic findings. Fourth, vendor-related variability in speckle-tracking software and strain measurement protocols may limit reproducibility across centers. Finally, while our follow-up duration of 5.6 years is notable, it may still be insufficient to capture the long-term incidence of clinically relevant outcomes, such as atrial fibrillation, particularly in athletes with prolonged training exposure over decades. Future longitudinal studies with broader cohorts and multimodal imaging are needed to confirm these findings and refine athlete-specific reference values.

## 5. Conclusions

In this long-term follow-up of professional soccer players, cardiac structure and function remained remarkably stable over a long period of time. LV and RV volumes, LV wall thickness, and strain values, as well as atrial size and reservoir function, remained within physiological ranges, with no indication of pathological remodeling. These results support the assumption that long-term high-level training in mixed sport disciplines, such as soccer leads to balanced, adaptive cardiac changes rather than subclinical disease.

## Figures and Tables

**Table 1:** Baseline characteristics.

Parameter	Baseline	End of Follow-Up	*p* Value
Age (years)	21.2 ± 3.4	26.8 ± 4.1	<0.001
Male (%)	20 (100)	-	-
Height (cm)	182.7 ± 6.1	183.0 ± 6.6	0.163
Weight (kg)	78.9 ± 6.7	80.2 ± 8.6	0.088
BSA (m^2^)	2.01 ± 0.12	2.02 ± 0.14	0.094
Heart rate (/min)	56.4 ± 9.8	60.1 ± 9.7	0.169
European origin (%)	13 (65%)	-	-
African origin (%)	7 (35%)	-	-

BSA = Body surface area.

**Table 2:** Echocardiographic parameters of left ventricular morphology and function.

Parameters	Baseline	End of Follow-Up	*p* Value
IVSD (mm)	9.9 ± 1.0	9.9 ± 1.2	0.878
LVPWD (mm)	9.7 ± 1.3	9.8 ± 1.7	0.707
LVEDD (mm)	54.5 ± 3.1	54.6 ± 3.9	0.868
RWT	0.35 ± 0.05	0.36 ± 0.09	0.539
LVMi (g/m^2^)	104.8 ± 16.6	101.5 ± 17.5	0.507
GLS (%)	−18.7 ± 2.2	−18.4 ± 1.9	0.670
D_LVOT_ (mm)	23.0 ± 1.2	23.9 ± 1.5	0.001
VTI_LVOT_ (cm)	21.9 ± 2.8	22.9 ± 2.8	0.067
LVSVi_Doppler_ (mL/m^2^)	44.9 ± 5.6	50.1 ± 5.0	<0.001
Cardiac Index (L/min/m^2^)	2.5 ± 0.6	3.0 ± 0.5	0.006
LVEDVi_biplane_ (mL/m^2^)	80.3 ± 8.6	78.3 ± 9.5	0.398
LVESVi_biplane_ (mL/m^2^)	34.1 ± 6.5	30.1 ± 7.1	0.064
LVSVi_biplane_ (mL/m^2^)	48.2 ± 11.6	48.2 ± 5.1	0.979
LVEF_biplane_ (%)	57.8 ± 5.4	61.9 ± 5.6	0.032
MAPSE (mm)	13.0 ± 3.0	12.8 ± 2.4	0.746
LVEDVi_3D_ (mL/m^2^)	82.2 ± 11.1	83.5 ± 12.2	0.982
LVESVi_3D_ (mL/m^2^)	36.3 ± 9.7	33.1 ± 10.6	0.157
LVSVi_3D_ (mL/m^2^)	45.9 ± 5.8	46.2 ± 12.3	0.715
LVEF_3D_ (mL/m^2^)	56.3 ± 7.5	58.6 ± 4.2	0.218

IVSD = interventricular septal thickness in diastole; LVPWD = left ventricular posterior wall thickness in diastole; LVEDD = left ventricular end-diastolic diameter; RWT = relative wall thickness; LVMi = left ventricular mass index; GLS = global longitudinal strain; D_LVOT_ = left ventricular outflow tract diameter; VTI_LVOT_ = velocity time integral of the LVOT; LVSVi = left ventricular stroke volume index; LVEDVi = left ventricular end-diastolic volume index; LVESVi = left ventricular end-systolic volume index; LVEF = left ventricular ejection fraction; MAPSE = mitral annular plane systolic excursion.

**Table 3:** Echocardiographic parameters of diastolic function.

Parameters	Baseline	End of Follow-Up	*p* Value
E/A-Ratio	2.01 ± 0.50	1.75 ± 0.41	0.035
E/e’ septal	5.8 ± 0.9	5.5 ± 1.0	0.266
E/e’ lateral	4.5 ± 1.0	4.2 ± 0.8	0.278
e’ septal	0.14 ± 0.02	0.14 ± 0.02	0.948
e’ lateral	0.18 ± 0.03	0.18 ± 0.03	0.733
E Wave (m/s)	0.79 ± 0.16	0.73 ± 0.09	0.189
A Wave (m/s)	0.40 ± 0.09	0.43 ± 0.08	0.210
TR-Vmax (m/s)	2.3 ± 0.2	2.3 ± 0.2	0.828
sPAP (mmHg)	20.4 ± 3.7	20.5 ± 3.1	0.866
LA Strain CD (%)	−23.2 ± 3.8	−23.6 ± 6.0	0.909
LA Strain CT (%)	−8.9 ± 2.7	−10.3 ± 3.2	0.064
LA Strain R (%)	32.1 ± 5.3	33.9 ± 6.9	0.301
LA Vol_max_ (mL)	58.5 ± 9.9	62.8 ± 15.9	0.305
LAVi (mL/m^2^)	29.8 ± 5.2	31.1 ± 7.4	0.401

TR-Vmax = maximum tricuspid regurgitation velocity; sPAP = systolic pulmonary arterial pressure; LA Strain CD = left atrial conduit strain; LA Strain CT = left atrial contractile strain; LA Strain R = left atrial reservoir strain; LA Vol_max_ = maximum left atrial volume; LAVi = left atrial volume indexed to body surface area.

**Table 4:** Echocardiographic parameters of right heart morphology and function.

Parameters	Baseline	End of Follow-Up	*p* Value
D_RVOT_ (mm)	23.2 ± 1.7	23.7 ± 1.7	0.096
VTI_RVOT_ (cm)	21.0 ± 2.7	21.7 ± 2.3	0.185
RVSVi_Doppler_ (mL/m^2^)	43.3 ± 6.7	47.1 ± 4.4	0.037
RA Strain CD (%)	−23.9 ± 5.6	−20.6 ± 7.3	0.085
RA Strain CT (%)	−8.1 ± 4.9	−9.4 ± 4.4	0.394
RA Strain R (%)	32.2 ± 8.5	30.0 ± 7.8	0.352
RA Vol_max_ (mL)	53.9 ± 18.0	54.6 ± 13.0	0.732
RAVi (mL/m^2^)	26.7 ± 8.5	26.9 ± 6.2	0.803
RV Free Wall Strain (%)	−17.9 ± 2.2	−18.5 ± 3.2	0.801
RVEDVi_3D_ (mL/m^2^)	84.8 ± 14.3	80.6 ± 29.8	0.401
RVESVi_3D_ (mL/m^2^)	40.4 ± 11.1	37.9 ± 15.5	0.574
RVSVi_3D_ (mL/m^2^)	44.3 ± 5.7	47.4 ± 5.7	0.671
RVEF_3D_ (%)	53.0 ± 6.7	53.3 ± 5.3	0.988
TAPSE (mm)	22.6 ± 3.3	23.3 ± 4.1	0.497

D_RVOT_ = right ventricular outflow tract diameter; VTI_RVOT_ = velocity time integral of the RVOT; RVSVi = right ventricular stroke volume index; RA Strain CT = right atrial contractile strain; RA Strain R = right atrial reservoir strain; RA Vol_max_ = maximum right atrial volume; RAVi = right atrial volume indexed to body surface area; RVEDVi = right ventricular end-diastolic volume index; RVESVi = right ventricular end-systolic volume index; RVSVi = right ventricular stroke volume index; RVEF = right ventricular ejection fraction; TAPSE = tricuspidal annular plane systolic excursion.

## Data Availability

All data supporting reported results can be found in the manuscript.
